# The Infra-Bullar Groove: Assessing a Novel Surgical Landmark for Identifying the Natural Maxillary Ostium

**DOI:** 10.3390/life16030475

**Published:** 2026-03-15

**Authors:** Jameel Ghantous, Ayalon Hadar, Itay Chen, Chanan Shaul, Boaz Forer

**Affiliations:** Department of Otolaryngology and Head and Neck Surgery, Shaare-Zedek Medical Center, Faculty of Medicine, Hebrew University of Jerusalem, Jerusalem 91031, Israel

**Keywords:** functional endoscopic sinus surgery, paranasal sinuses, natural maxillary ostium, surgical landmark, chronic sinusitis

## Abstract

Functional endoscopic sinus surgery (FESS) is the gold-standard surgical treatment for chronic rhinosinusitis (CRS) not responding to appropriate medical therapy. Identifying the natural maxillary ostium (NMO) during FESS is often challenging due to the lack of definitive landmarks. To aid in the identification of the NMO, we describe and assess the feasibility of using a new surgical landmark, the infra-bullar groove (IBG), and evaluate its visibility and reproducibility during FESS. **Methods:** Video recordings of 41 maxillary antrostomy procedures in patients with varying severity of CRS were reviewed. Surgeons of different experience levels assessed IBG visibility and its termination at the NMO. **Results:** In video recordings where the ethmoid bulla was preserved during uncinectomy, the IBG and its connection to the NMO were successfully identified by all reviewers in 100% of analyzable cases. The mean time to IBG identification did not significantly differ among surgeons. The IBG was consistently more pronounced in cases with well-pneumatized bullae. **Conclusions:** Under controlled surgical conditions where the ethmoid bulla is preserved during uncinectomy, the IBG demonstrates high visibility and reproducibility for locating the NMO. However, further prospective studies are needed to establish its real-world utility and impact on surgical outcomes.

## 1. Introduction

Endoscopic sinus surgery (ESS) has revolutionized the treatment of chronic rhinosinusitis (CRS) and has become the gold-standard surgical intervention for patients who fail to respond to appropriate medical therapy [1,2,3]. The primary objectives of this surgery include the enlargement of sinus ostia, the restoration of adequate sinus aeration, the enhancement of mucociliary transport, and the provision of an improved pathway for topical therapies [3,4,5]. The success of ESS is fundamentally dependent on precise anatomical knowledge and the consistent use of reliable anatomical landmarks. These landmarks serve as the surgeon’s navigational framework, ensuring safe and effective surgery while reducing the risk of complications and significantly lowering the chances of surgical failure.

Despite advances in endoscopic techniques and imaging technology, the intraoperative identification of critical anatomical structures remains challenging in many clinical scenarios. In real-life surgical situations, typical landmarks can become obscured or distorted by bleeding, mucosal edema, or extensive polyposis. Under these suboptimal visualization conditions, even experienced surgeons may encounter difficulty with anatomical orientation, while less experienced surgeons face substantially greater challenges in confidently identifying key structures. The inflammatory process itself can fundamentally alter normal anatomical relationships, creating a surgical field that bears little resemblance to textbook descriptions or preoperative imaging [6].

A critical and foundational step in ESS is the middle meatal antrostomy, typically the first and most frequently performed maneuver during functional sinus surgery. The accurate identification and appropriate enlargement of the natural maxillary ostium (NMO) are essential for successful surgical outcomes, as they ensure proper drainage and ventilation of the maxillary sinus while allowing for effective delivery of postoperative topical therapies. However, one of the most common and consequential surgical errors during this step is the inadvertent creation of an iatrogenic opening in the medial maxillary wall or the mistaken enlargement of an accessory ostium, leaving the true NMO unopened. This error, which can occur even in experienced hands, leads to a phenomenon known as “recirculation,” where secretions drain from the maxillary sinus through the surgically created opening only to be drawn back into the sinus through the unopened natural ostium. This perpetuates the cycle of inflammation, impairs normal ventilation and drainage, and ultimately contributes to persistent symptoms and surgical failure [1,2,6,7,8,9,10].

The difficulty in identifying the NMO is compounded by the frequent presence of accessory ostia in the medial wall of the maxillary sinus. These naturally occurring openings can be difficult to distinguish from the true NMO, particularly in the setting of inflammatory disease, where mucosal edema and polyps further obscure the anatomy. To date, despite the critical importance of accurately locating the NMO, no established anatomical landmark reliably assists surgeons in this task or in distinguishing the natural ostium from accessory openings. This represents a significant gap in our surgical armamentarium, as failure to identify and enlarge the true NMO can lead to persistent disease and necessitate revision surgery [4,11].

Embryologically, the maxillary sinus develops from an evagination of the lateral nasal wall during the third to fourth fetal month, with the natural ostium representing the original site of this outpouching into the ethmoid infundibulum [12,13]. The ethmoid bulla develops as the largest anterior ethmoid cell and pneumatizes inferiorly and anteriorly, creating a consistent anatomical relationship with the medial maxillary wall. The junction between the floor of the ethmoid bulla and the medial maxillary sinus wall forms a natural indentation or groove as these two bony structures meet at different developmental and pneumatization planes. This anatomical relationship is preserved across different degrees of bullar pneumatization, although the prominence of the groove may vary with the extent of inferior bullar extension [10,14].

**Figure 1:**
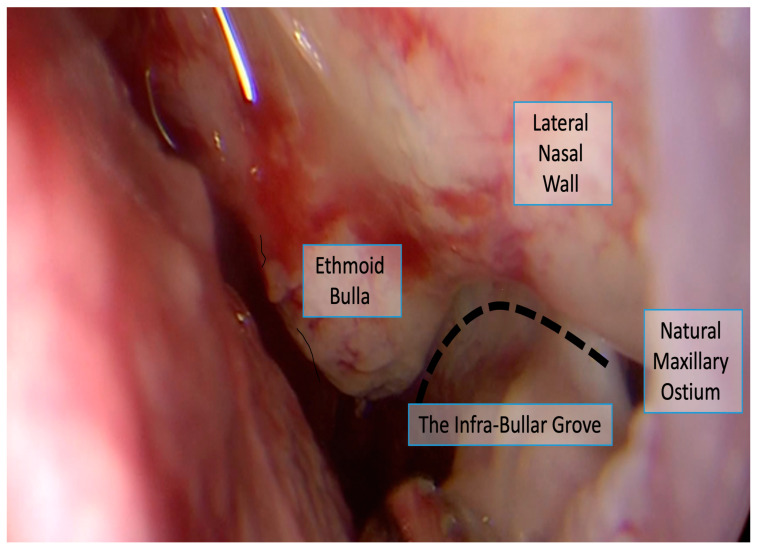
The infra-bullar groove is located at the junction of the ethmoid bulla floor and the medial maxillary wall; the NMO is located in its anterior part.

**Figure 2:**
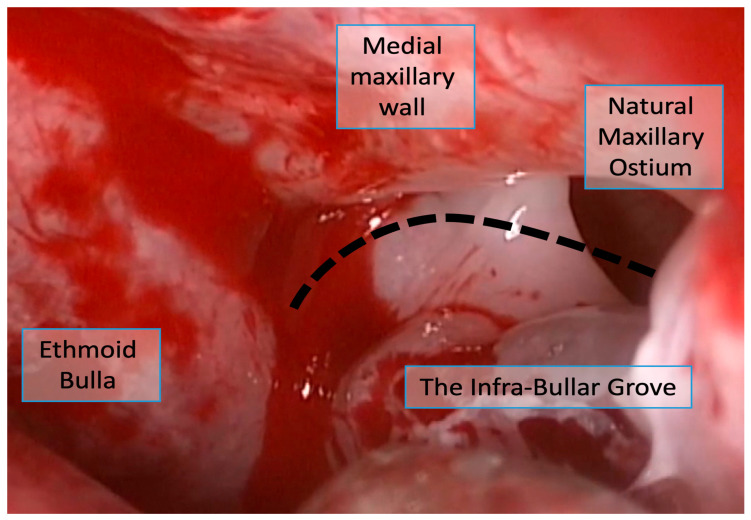
The infra-bullar groove in a patient with moderate CRS.

**Figure 3:**
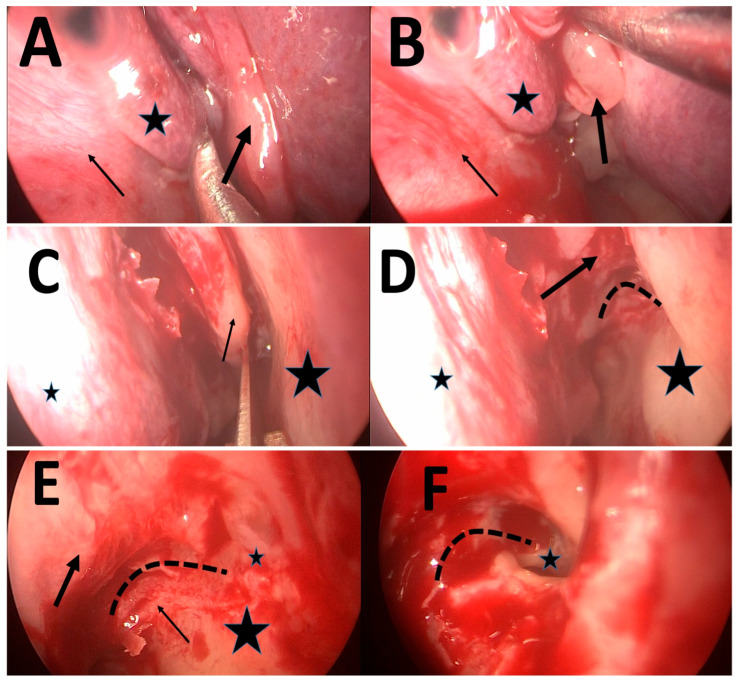
Left nasal cavity at the beginning of surgery in severe CRSwNP. (**A**,**B**) The nasal septum (thin arrow), middle turbinate (asterix), and polyps (thick arrow) can be seen. (**C**,**D**) A closer view of the left middle meatus after polyp removal. The middle turbinate (small asterisk) and lateral nasal wall (large asterisk) can be seen. (**C**) The uncinate process (thin arrow) is rotated anteriorly by the mass effect of the polyps is resected using a 15-blade scalpel. (**D**) Following uncinate removal, an edematous IBG (fine line) can be seen at the junction of the bullar floor (thick arrow) and lateral nasal wall (large asterix). (**E**,**F**) A closer view of the IBG using a 70-degree endoscope. (**E**) The edematous IBG (fine line) is present at the junction of the bullar floor (thick arrow) and lateral nasal wall (large asterix), the remnant uncinated bone (thin arrow) is still present, obstructing clear view of the IBG, and a small asterix is the anticipated location of the natural maxillary ositum. (**F**) After complete uncinectomy, the IBG (dotted line) leading to the natural maxillary ostium (small asterix) can be clearly seen.

To address this critical clinical need, we describe and assess the feasibility of using a simple and potentially identifiable surgical landmark: the infra-bullar groove (IBG). The IBG is a shallow indentation that becomes apparent at the junction of the ethmoid bulla floor and the medial maxillary wall. This groove becomes visible immediately after resection of the inferior portion of the uncinate process, a step routinely performed early in the surgical procedure. By carefully following this groove anteriorly with either a straight or angled endoscope, the surgeon may be guided to the location of the NMO (Figure 1, Figure 2 and Figure 3). The IBG thus potentially provides a surgical “roadmap” to orient surgeons during the critical phase of identifying and enlarging the natural ostium.

This study aimed to systematically describe this surgical landmark and assess its visibility and reproducibility in assisting surgeons in identifying the NMO under controlled surgical conditions. We sought to address several key questions regarding the feasibility and practical applicability of the IBG. First, we aimed to estimate the percentage of cases in which the IBG can be clearly identified during ESS across the spectrum of disease severity, including mild, moderate, and severe chronic rhinosinusitis, when the surgical technique preserves the ethmoid bulla during uncinectomy. Second, we examined whether the IBG consistently terminates anteriorly at the NMO in video recordings where it could be identified. Finally, we compared the ease of IBG identification among surgeons with varying levels of experience, ranging from junior residents in the early stages of training to senior rhinologists with extensive surgical experience.

## 2. Materials and Methods

### 2.1. Study Design and Patient Selection

This retrospective observational study was conducted at Shaare Zedek Medical Center, a tertiary medical center. The study was approved by the institutional review board with a waiver of informed consent due to its retrospective nature and the use of de-identified video recordings. Sequential video recordings of endoscopic sinus surgery procedures performed over five years were retrieved from the surgical database. Only the maxillary antrostomy portion of each procedure was extracted and edited for analysis. Patients were stratified into four groups: Group 1 consisted of patients undergoing ESS as part of transsphenoidal pituitary surgery with normal sinonasal anatomy, while Groups 2–4 comprised patients with chronic rhinosinusitis categorized by the Lund–Mackay CT score as mild (LMS < 12), moderate (13 ≤ LMS < 18), or severe (19 ≤ LMS ≤ 24) disease.

Inclusion criteria were primary ESS procedures in patients aged 18 years or older with adequate video quality for anatomical assessment. Exclusion criteria included revision ESS cases, significant intraoperative bleeding obscuring visualization, cases where the ethmoid bulla was opened prior to uncinectomy completion, and poor video quality precluding anatomical identification.

### 2.2. Sample Size Justification

This exploratory study aimed to assess the feasibility and reproducibility of identifying a novel surgical landmark across different disease severities and surgeon experience levels. Based on similar validation studies of surgical landmarks in endoscopic sinus surgery [14,15], a sample size of approximately 40 cases was determined to be sufficient for preliminary assessment of identification rates and inter-rater reliability. The final analysis of 41 video clips provided representation across mild (n = 14), moderate (n = 13), and severe (n = 14) CRS categories.

Surgical Technique. All procedures followed a standardized protocol. After general anesthesia induction, topical decongestion was achieved using cotton pledgets soaked in adrenaline 1:3000 and cocaine 4%. The uncinate process was resected using a 15-blade scalpel or through-cutting instruments while preserving the ethmoid bulla. In cases with significant infundibular edema, careful mucosal removal was performed while preserving the bullar walls to expose the bullar floor attachment to the lateral nasal wall. The IBG was identified as a shallow indentation at the junction between the ethmoid bulla floor and the medial maxillary wall, then traced anteriorly until terminating at the natural maxillary ostium. Middle meatal antrostomy was then performed by enlarging the ostium while preserving surrounding structures. Visualization was performed with 0° and 70°-angled endoscopes.

### 2.3. Evaluation Protocol

Three surgeons with varying levels of surgical experience served as independent, blinded reviewers: a senior rhinology surgeon, a senior ENT resident in final-year training, and a junior ENT resident in second-year training. Before formal evaluation, all surgeons underwent standardized training using video clips from Group 1 patients to familiarize them with IBG anatomy and its relationship to the NMO. Following training, each surgeon independently reviewed all video recordings from Groups 2–4 in randomized order. For each case, reviewers documented the time point of the first IBG identification relative to the video clip onset and whether the groove led to the NMO using a standardized data collection form.

All reviewers were fully blinded to clinical information. Video clips were provided without accompanying clinical data, including diagnosis, disease severity classification, Lund–Mackay CT scores, patient demographics, or preoperative imaging findings. Videos were presented in random order, and reviewers were blinded to one another’s assessments. To approximate real-time intraoperative conditions as closely as possible in a video-based study, reviewers were given strict viewing constraints. Specifically, reviewers were instructed NOT to pause, rewind, or replay any portion of the video clips during assessment. Each video was viewed once, continuously, from beginning to end, requiring real-time anatomical identification without the opportunity for repeated examination. This protocol was designed to simulate the temporal constraints of actual surgery, in which anatomical structures must be identified dynamically without the ability to pause or review.

Similarly, the training protocol for resident reviewers was deliberately limited. Training consisted of brief verbal explanation of the IBG concept followed by single-view presentation of example cases from skull base surgeries showing healthy sinus anatomy. Residents were not permitted to review training videos multiple times.

### 2.4. Outcomes and Statistical Analysis

The primary outcome was time to IBG identification, measured from the onset of the edited video clip until the reviewer indicates groove identification. Secondary outcomes included the IBG identification success rate across disease severity groups, the correlation between IBG and the natural maxillary ostium location, and inter-observer agreement in identification time.

Paired *t*-tests compared mean identification times across reviewers with different levels of experience. The intraclass correlation coefficient for absolute agreement assessed consistency in identification times across all reviewers. All analyses were performed using SPSS Statistics Version 25 (IBM Corp., Armonk, NY, USA). Continuous variables are reported as the mean ± standard deviation, with *p* < 0.05 considered statistically significant.

### 2.5. Data Management

All video clips were de-identified before review to maintain patient confidentiality. Each clip was assigned a unique identifier to facilitate blinded review.

## 3. Results

### 3.1. Patient Selection and Video Analysis

Thirty patients with chronic rhinosinusitis undergoing primary endoscopic sinus surgery were initially identified from the surgical database. Video recordings of bilateral maxillary antrostomy procedures yielded 60 video clips after extraction and editing. During quality control review, 19 clips (31.7%) were excluded from analysis. Twelve clips (20.0%) were excluded due to significant intraoperative bleeding that obscured visualization of the surgical field and prevented adequate assessment of the infra-bullar groove. Seven clips (11.7%) were excluded because the ethmoid bulla was dissected before completion of the uncinectomy, thereby altering the normal anatomical landmarks.

**Figure 4:**
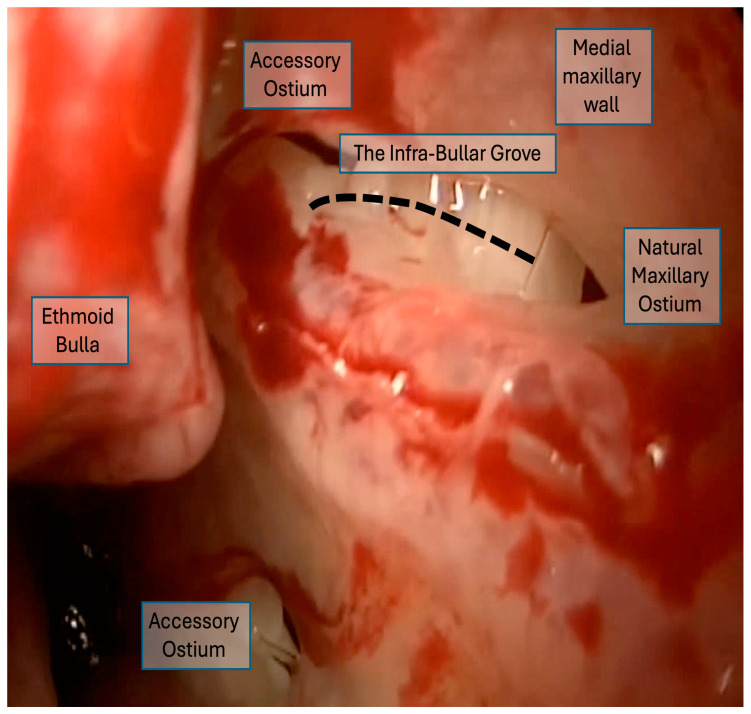
A 70° endoscope view of the left nasal cavity after uncinectomy. Three openings are visible in the medial wall of the maxillary sinus, which can create confusion in identifying the natural maxillary ostium (NMO). Using the infra-bullar groove (IBG) as a landmark confirms that the true NMO is located in the posterior part of the groove. At the same time, the anterior opening represents an %accessory ostium and cannot be the NMO.

The final analysis included 41 video clips representing 41 maxillary sinuses from 21 patients (Figure 1). The distribution by disease severity was as follows: 14 sinuses (34.1%) from 7 patients with mild CRS (Lund–Mackay score < 12), 13 sinuses (31.7%) from 7 patients with moderate CRS (Lund–Mackay score 13–18) (Figure 2), and 14 sinuses (34.1%) from 7 patients with severe CRS (Lund–Mackay score 19–24) (Figure 3 and Figure 4). All video clips were reviewed independently by three surgeons with varying levels of experience, yielding a total of 123 independent assessments.

### 3.2. Infra-Bullar Groove Identification

Among the 41 video recordings that met the inclusion criteria after the quality control review (representing 68.3% of the initially identified cases), all three reviewers successfully identified the IBG in all cases, yielding a 100% identification rate (41/41 per reviewer, 123/123 total assessments). This identification success rate was consistent across all disease severity categories. It is important to note that this identification rate applies only to carefully selected and analyzable video recordings. A substantial proportion of cases (31.7%) were excluded due to significant bleeding or premature bulla dissection, representing precisely the challenging scenarios where landmark identification would be most clinically valuable. While our analysis included cases across the spectrum of disease severity—mild (34.1%), moderate (31.7%), and severe (34.1%) CRS—cases requiring exclusion due to extreme conditions demonstrate that the IBG, like other anatomical landmarks, may not be consistently identifiable in all surgical scenarios. In all 123 assessments of analyzable video recordings in which the IBG was identified, reviewers confirmed that tracing the groove anteriorly directly led to the NMO. No instances were recorded where the IBG led to an accessory ostium or failed to terminate at the NMO.

### 3.3. Time to Identification

The mean time from video clip onset to IBG identification for each reviewer is presented in Table 1.

Paired *t*-test comparisons of mean identification times between reviewers showed no statistically significant differences. The comparison between the senior surgeon and senior resident yielded a mean difference of 29 s (*p* = 0.110). The comparison between the senior surgeon and junior resident showed a mean difference of 6 s (*p* = 0.727). The comparison between the senior resident and junior resident demonstrated a mean difference of −23 s (*p* = 0.052).

### 3.4. Inter-Rater Reliability

The intraclass correlation coefficient for absolute agreement across all three reviewers was 0.961 (95% CI: 0.932–0.979, F-test (40,82) = 50.8 *p* < 0.001), indicating high consistency in identification times across reviewers. This analysis included all 41 cases with measurements from all three reviewers.

## 4. Discussion

This study describes and assesses the feasibility of using the IBG as a surgical landmark for identifying the NMO during ESS. Our findings demonstrate that, under controlled conditions in which the ethmoid bulla is preserved during uncinectomy and adequate visualization is maintained, the IBG can be reliably identified by surgeons of all experience levels, with a 100% identification success rate across 41 analyzable cases. These results demonstrate feasibility under controlled video review conditions in carefully selected cases. Furthermore, in all cases where the IBG was identified, it consistently led to NMO, with no instances of misidentification or termination at accessory ostia. These results suggest that the IBG represents the surgically revealed junction where the floor of the ethmoid bulla meets the medial wall of the maxillary sinus. The IBG may represent a potentially useful surgical guide under specific surgical conditions, although important limitations regarding its universal applicability must be acknowledged.

The introduction of novel anatomical terms requires consideration of existing international nomenclature. The Terminologia Anatomica (TA2), maintained by the Federative International Programme for Anatomical Terminology (FIPAT), provides standardized anatomical nomenclature [15]. A review of TA2 terminology reveals that while specific terms exist for the ethmoid bulla (bulla ethmoidalis) and maxillary ostium (ostium maxillare), no current term specifically describes the groove formed at their junction. The term “infra-bullar groove” (sulcus infrabullaris) is proposed as a descriptive clinical term denoting the indentation inferior to the ethmoid bulla where it meets the medial maxillary wall. However, formal recognition of this term would require cadaveric anatomical studies, morphometric validation, and eventual submission to FIPAT for consideration, which represents an important direction for future research.

We conducted a comprehensive historical literature review, including PubMed/MEDLINE (no date restrictions), historical ESS texts [16,17], anatomical atlases, Terminologia Anatomica, and citation analysis. This did not identify prior description of the bullar floor–maxillary wall junction as a surgical landmark for NMO identification. However, we acknowledge our search may not have been exhaustive; this relationship may have been recognized informally or described using different terminology in non-English research. We cannot definitively rule out rediscovery and welcome any references to prior descriptions.

The clinical significance of these findings lies in their potential to address a fundamental challenge in ESS. Despite decades of experience with ESS, accurate identification of the NMO remains problematic. Studies examining revision ESS have identified incomplete uncinectomy and failure to include the NMO in the surgical antrostomy as among the most common causes of surgical failure [18]. A systematic review of anatomical landmarks in navigated ESS identified seven critical landmarks—the maxillary sinus ostium, orbital wall, frontal recess, skull base, ground lamella, fovea posterior, and sphenoid sinus ostium—yet acknowledged that approximating the NMO’s position during anterograde surgery remains challenging [19]. Recently, the transverse turbinate line (TTL) was proposed as a landmark providing craniocaudal positioning for the NMO [14]. The IBG may complement this approach by offering an anteroposterior landmark that becomes visible after uncinectomy and can be traced directly to the NMO, even in cases with severe inflammatory disease, where traditional anatomical relationships are obscured [20].

The challenge of landmark identification in severe inflammatory conditions is not unique to the IBG. Well-established anatomical landmarks—including the lamina papyracea, fovea ethmoidalis, natural sphenoid ostium, and the natural maxillary ostium itself—are also difficult to identify during challenging surgery in severe chronic rhinosinusitis with nasal polyposis (CRSwNP). The presence of extensive polyposis, severe mucosal edema, or active bleeding compromises visualization of all anatomical reference points, yet these traditional landmarks remain valid and clinically valuable when conditions permit their identification. Similarly, the IBG should be evaluated as a potential adjunct reference point when adequate visualization is achieved, not as a universal solution for all surgical scenarios. Our proof-of-concept study demonstrates that when visualization is adequate and surgical technique preserves bullar anatomy, the IBG can be reliably identified across a spectrum of disease severity, including in a substantial proportion of severe cases (34.1% of our included cases represented severe CRS).

In the context of existing approaches, several anatomical landmarks have been described for NMO localization, each with specific advantages and limitations. The uncinate process serves as the primary landmark, with the NMO typically located posterosuperior to its attachment; however, extensive inflammation or anatomical variants can obscure this relationship [12,21]. The lacrimal bone and nasolacrimal duct provide superior reference points, but their identification requires familiarity with the orbital anatomy and may not be readily visible in all surgical approaches [22]. The fontanelle region posterior to the uncinate is another common reference, though accessory ostia in this area can lead to confusion [23]. The recently described TTL offers a vertical reference, intersecting the NMO in most cases, but does not provide anteroposterior guidance [14]. In contrast, the IBG offers a dynamic landmark that becomes visible after uncinectomy and provides a direct anatomical path to the NMO by following the natural junction between ethmoid and maxillary structures. However, this advantage is contingent upon maintaining bulla integrity and adequate visualization, conditions that may not be achievable in all clinical scenarios.

Beyond the choice of landmark, an interesting observation in our video-based assessment was that reviewers with different levels of surgical experience—ranging from a second-year ENT resident to a senior rhinology surgeon—could identify the IBG in single-view video clips under constrained viewing conditions (no pausing or replaying). While reviewers were instructed to approximate real-time viewing, we acknowledge that video review—even with temporal constraints—cannot fully replicate the cognitive load, instrument handling, active bleeding, and time pressure of actual surgery. Therefore, while these findings suggest that the IBG may be identifiable across different experience levels, prospective evaluation in real-time surgical conditions is essential to determine whether this translates to actual intraoperative utility. This finding may have implications for surgical training, as studies on the ESS learning curve have consistently shown that spatial orientation—rather than manual dexterity—represents the primary challenge for trainees [24,25]. Eye-tracking studies in ESS training have demonstrated that residents require extensive practice to develop efficient gaze strategies and reduce cognitive load [25], while simulation-based training requires multiple sessions to achieve plateau performance [26]. The high intraclass correlation coefficient (0.961) provides evidence for the reproducibility of IBG identification in postoperative video review, though this may not translate to real-time surgical performance.

The IBG represents the surgically revealed junction where the floor of the ethmoid bulla meets the medial wall of the maxillary sinus. This confluence of bony structures creates an anatomical relationship that may be preserved despite variations in bulla size and pneumatization patterns. Our qualitative observations suggest that the prominence of the IBG may be enhanced in cases with extensive bullar pneumatization, particularly when the bulla extends below the level of the orbital floor. However, even in cases with minimal bullar pneumatization among the analyzable videos, the groove remained identifiable after proper uncinectomy. While image-guided navigation systems have improved safety in complex ESS, their utility depends on registration accuracy and clear anatomical visualization [19]. The IBG could potentially be used both with and without navigation, serving as a confirmatory landmark for image-guided procedures and as a reference in non-navigated surgery, though this remains to be prospectively validated.

Supporting this anatomical hypothesis, the high inter-rater reliability (ICC 0.961) and consistent identification across all cases with varying disease severity—including mild (34.1%), moderate (31.7%), and severe (34.1%) chronic rhinosinusitis—suggest that a recognizable anatomical relationship exists at this location when adequate surgical exposure is achieved. The ease of identification during surgery across this spectrum of disease severity supports the existence of this surgically defined landmark. However, whether this represents a consistent underlying bony structure or a technique-dependent visual feature requires formal anatomical validation through cadaveric dissection, morphometric analysis, and radiological correlation.

These findings have potential educational implications. Recent advances in surgical education emphasize the importance of validated anatomical landmarks in improving trainee confidence and reducing complications [24,25]. Virtual reality simulation and augmented reality platforms are increasingly incorporating anatomical landmarks to enhance spatial understanding [22,26]. If validated through prospective real-time surgical studies, the IBG could potentially be integrated into these educational tools. However, such integration would require demonstrating clinical utility beyond the controlled conditions of this videoreview study.

Regarding anatomical consistency, an important observation in our study was the absence of apparent variation in IBG location or configuration across the 41 analyzed cases, despite diversity in disease severity and bulla morphology. This consistency is analogous to the NMO, which maintains a stable anatomical position relative to the infundibulum, even though its visibility may be affected by surrounding anatomical variants or inflammatory changes. From an embryological perspective, this consistency is logical: the NMO and its surrounding structures, including the junction between the ethmoid bulla and maxillary sinus, develop together as an integrated anatomical unit [10,27]. Just as the NMO does not vary significantly in location despite variations in surrounding structures, the IBG appears to represent a stable junctional landmark. However, we must distinguish between variations affecting *visibility* versus variations in the *IBG itself*. Common anatomical variants such as concha bullosa, extensive bulla pneumatization, Haller cells, or aberrant uncinate attachments may affect the surgical approach and ease of IBG visualization without necessarily altering the fundamental anatomical relationship. This is similar to how these same variants may make the NMO more difficult to identify without changing its actual location.

While we observed no apparent variation in IBG location or configuration across our 41 cases, our study did not systematically categorize cases by specific anatomical variant subtypes (concha bullosa, Haller cells, paradoxical middle turbinate, etc.). Although the IBG appeared consistently present across diverse bulla morphologies and disease severities in our sample, formal anatomical validation through larger-scale cadaveric studies is needed to definitively determine whether the IBG maintains consistent characteristics across the full spectrum of sinonasal anatomical variations. Our sample size may not capture rare anatomical configurations that could affect IBG characteristics.

Despite this apparent consistency, a crucial safety consideration is whether mucosal edema could create ‘false landmarks’ that superficially resemble the IBG. Based on the pathophysiology of mucosal edema, this appears unlikely—when mucosa becomes edematous, it expands and fills anatomical depressions, making underlying grooves *less* visible rather than creating new apparent grooves. This is supported by our experience in skull base surgery cases with minimal inflammatory edema; in such cases, the IBG is consistently identifiable, supporting that it represents a true bony anatomical relationship. The clinically relevant concern is that severe inflammation may *obscure* the true IBG, making it unidentifiable—similar to how severe edema obscures other established anatomical landmarks such as the NMO itself, the lamina papyracea, and the fovea ethmoidalis. As with all anatomical references in ESS, the IBG should be used as an adjunct to—not a replacement for—comprehensive anatomical assessment, including confirmation of underlying bony structures, palpation when feasible, and correlation with preoperative imaging.

This study has several important limitations that must be carefully considered. Most significantly, there is an absence of formal anatomical validation through cadaveric dissection, morphometric studies, or radiological correlation; we cannot confirm whether the IBG represents a consistent bony anatomical structure present across different populations and anatomical variants without such studies. The retrospective design and reliance on edited video recordings introduce substantial methodological limitations, as video review permits repeated viewing without the cognitive and technical demands of real-time surgery, likely overestimating the ease of IBG identification in actual clinical practice. All surgeries were performed using a specific technique where the ethmoid bulla was deliberately preserved during uncinectomy, limiting applicability to surgeons who routinely perform early bullectomy. Furthermore, all reviewers underwent standardized training before evaluation, introducing confirmation bias, as the 100% identification rate may reflect this prior training rather than the inherent visibility of the structure.

Moreover, the 31.7% exclusion rate has important implications. These excluded cases, particularly those with significant bleeding or that require an altered surgical approach, represent precisely the challenging scenarios in which a reliable landmark would be most clinically valuable. The high exclusion rate indicates that our findings apply only to a subset of surgical cases in which optimal visualization can be maintained and the standard surgical sequence can be followed. This substantially limits the generalizability of our observations and underscores that the IBG may be neither identifiable nor reliable in approximately one-third of real-world surgical scenarios.

Beyond the exclusion rate, additional limitations include the small sample size (41 video clips from 21 patients) and single-center design, which restricts generalizability to diverse surgical practices and patient populations. Only three reviewers from a single institution participated, limiting the diversity of surgical experience and training backgrounds represented. While we demonstrate that the IBG can be identified in edited videos under controlled conditions, we cannot claim that using this landmark improves patient outcomes or reduces complications. The study was limited to primary ESS procedures, with no assessment of the IBG’s utility in revision surgery, where anatomical landmarks are typically distorted.

While we implemented strict viewing constraints—specifically prohibiting pausing, rewinding, or replaying video clips—to approximate real-time surgical conditions, important methodological differences remain between our video-based assessment and actual intraoperative landmark identification. Video review, even under temporal constraints, eliminates the cognitive load of simultaneous instrument manipulation and surgical decision-making. The edited videos excluded segments with significant active bleeding or poor visualization, precisely the conditions where landmark identification would be most challenging in live surgery. Additionally, reviewers were aware that this was a study assessment rather than patient care, which may have reduced the psychological pressure and time constraints inherent in actual surgery. Therefore, while our no-pause protocol provides a closer approximation to real-time conditions than traditional video review studies, our findings must be validated in prospective intraoperative studies before conclusions about surgical utility can be drawn.

Given these limitations, several critical areas for future research can be defined. First and most importantly, formal anatomical validation through cadaveric dissection and morphometric studies is essential to determine whether the IBG represents a consistent anatomical structure across diverse populations. Second, prospective studies evaluating the real-time application of the IBG during live surgery are needed to assess its practicality. Finally, multicenter studies involving surgeons from diverse training backgrounds would help establish generalizability.

## 5. Conclusions

This exploratory study describes the IBG as a potential surgical landmark for identifying the NMO during ESS and demonstrates its visibility and reproducibility. Surgeons with varying levels of experience can identify the IBG in edited video recordings with high consistency. In the analyzable cases, the groove consistently showed a relationship with the NMO. These findings should be interpreted as preliminary evidence of feasibility and reproducibility under controlled and specific circumstances and cannot be generalized to all clinical scenarios, particularly those with significant bleeding or anatomical distortion that have been shown to prevent landmark assessment in approximately one-third of initially screened cases. Substantial additional research, including cadaveric anatomical studies, prospective real-time surgical evaluation, and clinical outcome assessment, is essential before the IBG can be recommended for routine clinical practice. If future studies confirm these preliminary observations and demonstrate clinical benefit, the IBG may eventually prove to be a useful adjunct to existing anatomical landmarks in ESS.

## Figures and Tables

**Table 1:** Time to Infra-Bullar Groove Identification by Reviewer Experience Level. Statistical Comparisons Between Reviewers.

Reviewer	Mean Time (min:sec)	Standard Deviation	Success Rate
Junior Resident	4:48	±3:20	41/41 (100%)
Senior Resident	4:25	±3:46	41/41 (100%)
Senior Surgeon	4:54	±3:21	41/41 (100%)
Overall	4:42	±3:29	123/123 (100%)

## Data Availability

The datasets used and analyzed during the current study are available from the corresponding author upon reasonable request, subject to privacy and ethical restrictions.

## Abbreviations

The following abbreviations are used in this manuscript:

FESS	Functional endoscopic sinus surgery
CRS	Chronic rhinosinusitis
NMO	Natural maxillary ostium
IBG	Infra-bullar groove
